# Lived Experience, Social Support, and Challenges to Health Service Use during the COVID-19 Pandemic among HIV Key Populations in Indonesia

**DOI:** 10.21203/rs.3.rs-3282353/v1

**Published:** 2023-09-07

**Authors:** Evi Sukmaningrum, Judith Levy, Made Diah Negara, Devika N/A, Brigitta Dhyah K Wardhani, Luh Putu Lila Wulandari, Pande Putu Januraga

**Affiliations:** Faculty of Psychology, Atma Jaya Catholic University of Indonesia; University of Illinois at Chicago; University Centre of Excellence-AIDS Research Centre Health Policy and Social Innovation, Atma Jaya Catholic University of Indonesia; University Centre of Excellence-AIDS Research Centre Health Policy and Social Innovation, Atma Jaya Catholic University of Indonesia; Center for Public Health Innovation, Faculty of Medicine, Udayana University; Kirby Institute, UNSW; Department of Public Health and Preventive Medicine, Faculty of Medicine, Udayana University

**Keywords:** COVID-19 Indonesia, HIV key populations, Effects of government COVID-19 policy, Social Support, Health services experience, Antiretroviral adherence

## Abstract

The COVID-19 has greatly affected the lives, health and social well-being of people globally including presenting special challenges in low to middle income countries for people living with HIV. This study investigates the pandemic experiences of the four key HIV-positive populations in Indonesia: men who have sex with men, transgender women, female sex workers, and people who use drugs. In-depth interviews were conducted with a convenience sample of 22 key population members recruited through 9 nongovernment HIV agencies in Jakarta and Bali, Indonesia. Indonesia’s Large-scale Social Restrictions Policy mandating physical distancing and stay-at-home orders had been in effect for 7–10 months. The interviews were audio-recorded, transcribed, and coded using NVivo^™^ (R1.7) software A grounded theory approach identified key concepts along with similarities, differences, and reoccurring patterns of COVID-related lived experience among participants. Participants recounted the impact of both the pandemic and the Restriction Policy on their interpersonal, financial, medical, and psychosocial well-being. When in need, they turned to formal and informal sources of financial and social support plus their own resourcefulness. Along with other factors, HIV medication shortages, HIV and COVID-related stigma, and fear of acquiring COVID-19 negatively impacted antiretroviral adherence and the use of health services. The results point to the latent consequences of government attempts to curb a pandemic through public health lockdowns and enforced policies of physical separation. Its findings reveal the importance of ensuring that public safety nets for HIV key populations are available to supplement more informal personal sources of needed support.

## Introduction

The COVID-19 pandemic has greatly disrupted many aspects of human life throughout the world while exerting a draconian effect on the social fabric, economy, and population health of many countries. Mounting mortality and morbidity rates of COVID-19 led multiple governments to enact large-scale restrictions on population mobility, group and mass gatherings, and face-to-face social and physical interaction. Legal mandates to observe physical distancing between individuals significantly altered “normal” human routines and redefined the nature of interpersonal relationships [1, 2].

Numerous studies have investigated the pandemic’s effects on the health and well-being of people who are HIV-positive [3]. Results show that the many structural, cultural, psychosocial, and health factors that surround and complicate the lives of people living with HIV (PLWH) have both produced and exacerbated the challenges of living within the psychosocial and physical environment imposed by the pandemic. Yet despite a growing body of global research in multiple countries around the globe, few studies appear to specifically examine the combined effect of HIV and COVID-19 on the life experiences and psychosocial well-being of PLWH in Southeast Asia. The few exceptions include research investigating personal access to health care and supportive services in Malaysia among PLWH who use drugs [4], HIV disclosure dilemmas among HIV-positive women seeking COVID-19 treatment in Sumatra, Indonesia [5], and COVID-related threats to health and well-being among vulnerable HIV groups in Vietnam [1].

Drawing on data collected through qualitative in-depth interviews, this paper addresses this existing gap in HIV science by examining the impact of the pandemic on the lived experience, social support, and challenges to health service use among of members of four HIV key populations in Indonesia during the first year of the country’s COVID-19-pandemic. Home to the world’s fourth largest population, Indonesia has experienced one of the highest rates of COVID-19 infections in the region since the start of the pandemic, and its number of COVID-19 deaths rank uppermost in Southeast Asia [6]. In addition, although Indonesia has seen a gradual reduction in annual HIV incidence since 2010, its estimated number of people dying from HIV-related medical causes continues [7]. From this syndemic standpoint, Indonesia offers a highly important and informative vantage from which to study the effects of two intersecting pandemics on PLWH.

Indonesia’s first case of COVID-19–19 was confirmed in March 2020. As of January 2023, more than 6 million people had been diagnosed with the virus and over 160,000 Indonesians had died [8]. The Indonesian government’s initial response to the country’s rising pandemic was to institute a Large-scale Social Restrictions Policy (*Pembatasan Sosial Berskala Besar*) in Jakarta, Bali and several other cities and provinces with mounting COVID-19 prevalence. The policy permitted essential offices and factories to remain open, but restricted the activities of nonessential firms [9]. It also allowed people to engage in religious and essential life activities within certain limitations while simultaneously mandating school closings, prohibiting most internal and international travel, reducing public transportation, and issuing stay-at-home requirements except for essential trips. As the outbreak worsened, the government implemented a subsequent series of mandates designed to further restrict community interaction and population mobility [9, 10].

Like other LMIC countries with limited health resources [11], Indonesia has struggled throughout the COVID-19 pandemic with little available money to keep its citizens healthy or to supplement the income of those facing financial destitution due to its effects. With a chronically understaffed workforce and underfunded infrastructure, the added demands of the pandemic placed an additional burden on Indonesia’s already vulnerable health care system and strained its pre-COVID-19 delivery of HIV medical services and treatment for PLWH [12].

It is within this highly challenged socio-economic context that this study investigates the COVID-19-related experiences of the four major HIV-positive key populations in Indonesia: men who have sex with men (MSM), transgender women (TGW), female sex workers (FSW), and people who use drugs (PWUD). In examining their experiences, the study helps to extend the slim body of non-clinical research that considers the dual effects of COVID-19 and HIV on PLWH in a South-East Asian country. The study’s inductive qualitive approach allows study participants to describe their experiences in their own way and in their own words. Their accounts provide insight into the latent consequences for PLWH of government attempts to curb infectious disease transmission through large-scale community lockdowns and social distancing mandates. Embedded in the participants’ words are clues as to social policy and intervention strategies that may be needed to assist PLWH as the COVID-19 pandemic recedes globally, but its negative psychosocial and economic residual effects continue in Indonesia and likely in other low-to-middle income countries and as new pandemics possibly emerge to take its place.

## Methods

The data were collected in October through December 2020 when Indonesia’s Large-scale Social Restrictions Policy had been in effect for approximately 7–10 months. Twenty-two participants were recruited in Jakarta and Bali, Indonesia through referral from 9 nongovernment agencies and other local community groups that specifically serve people living with HIV. To be eligible to participate in the study, participants had to be HIV-positive, a member of a key population (FSW, MSM, TGW, or PWUD), receiving ARV treatment, and willing to provide informed consent to be interviewed and audio-recorded.

In-depth interviews were conducted in Bahasa Indonesian by trained, native-speaking interviewers. Participants could select which of three interviewing methods that they preferred: by Zoom (either with video or audio only), WhatsApp (either with video or audio only) or telephone (audio only). Interview questions asked participants about their interpersonal, psychosocial, and practical experiences with COVID-19 during the government COVID-19 lock-down. The audio-taped interviews lasted from 50 to 120 minutes. Participants received 200.000 Indonesian Rupiah (about $13 USD) as compensation for their time and internet or phone costs. The study has been approved by the Human Research Ethics Committee of the Faculty of Medicine, Udayana University/Sanglah Hospital with ethical clearance number 1806/UN14.2.2.VII.14/LT/2020. Informed consent was obtained from all individual participants included in the study.

### Data Coding and Analysis

The audio-recorded interviews were transcribed verbatim and coded using NVivo^™^ software version R1.7. The data were then analyzed inductively using grounded theory[13] to identify key concepts along with similarities, differences, and reoccurring COVID-19-related experiences reported by participants. The coding process began by reading and rereading all interview transcripts to identify patterns and themes in the data related to the participants’ COVID-19 experiences. Three major experiential categories emerged out of the data: the impact of the pandemic and government lock-down policies on participants’ interpersonal, financial, medical, and psychosocial wellbeing; formal and informal sources of social support; and challenges related to health service use. Second level coding involved identifying clusters of experiences and patterns of behavior that fit under each of these three coding categories. These subcategories were checked against each other to identify both consensus and diversity among participants in their COVID-19 accounts.

## Results

Table 1 reports the demographic characteristic of participants categorized according to how they self-identified when seeking HIV services from one of the study’s seven referring organizations. Of the 22 participants, 9 (41%) self-identified as MSM, 5 (23%) as FSW, 4 (18%) as TGW, and 4 (18%) as PWUD. Most participants (59%) were between 29 to 39 years of age. Females, including 4 transwomen, made up nearly 41% of the sample. Of the 22 participants, only 4 (18%) were unemployed with the remainder working either as staff at an NGO, within the private sector, as small business entrepreneurs, or as commercial sex workers. Sixteen (70%) had attended or graduated from senior high school including 4 participants who had attended college. MSM reported the highest level of educational attainment of all four key population groups. Slightly more participants were from Jakarta (53%) than Bali.

### Impact of the Pandemic on the Lived Experience of Indonesia’s HIV Key Populations

#### Impact on financial security

All 22 respondents reported being under considerable financial stress due to a range of COVID-19-related income challenges that included potential or actual job loss, reduced working hours, loss of employment-related benefits, and/or job insecurity. A transgender woman explains about the financial challenges of becoming destitute due to COVID-19:

Since COVID-19–19, I lost one job, and my daily income decreased, not increased. In fact, I have nothing at all. There is no income, yet the rent has to be paid, the electricity has to be paid, the water has to be paid. (01, TGW, Jakarta)

Working strictly from home during the lock-down could offer participants working for agencies, schools, and small business firms the opportunity through the internet for continued employment, but not without income loss. NGO personnel, for example, complained of losing transportation and food allowances that were customary job entitlements. Pre-COVID-19, they had received monetary compensation above their basic salaries to cover meals, public transportation costs, and other expenses incurred when working in the community. Such compensation could be stretched to help offset minor nonemployment-related personal expenses. Now under COVID-19, they were solely reimbursed for home internet fees incurred when attending staff meetings or conducting community outreach work via zoom.

In terms of [income from] work, it has decreased because we received additional compensation for food and transportation for doing community outreach. When we are on WFI, we do not have that compensation anymore, and instead receive only small amounts for zoom meetings and for all kinds of online activities. So my salary has been much reduced and almost half of what it was before. (08, TGW, Jakarta)

Meanwhile, participants who engaged in commercial sex reported shrinking income as client demand diminished. The government lockdown, the rigors of maintaining physical distancing, and worry about acquiring COVID-19 discouraged or negatively redefined the prevalence and nature of sex-for-money exchanges. Nonetheless, the need to support themselves and/or their families could override initial hesitation to engage in commercial sex:

I didn’t lose my job. It’s just that there are not as many guests as before. Sometimes there are guests, sometimes only one guest every two days.” (07, FSW, Bali).

To be honest, the hardest thing was that most people wanted to have sex with me for free. Oh my gosh [I thought], I just need to eat while you want it for free. (01, TGW, Jakarta)

Financial need also drove participants to find alternative sources of income to cover the many expenses of daily life. These tactics included drawing on their savings and/or borrowing money from friends and family. As one participant sadly admitted, “Like it or not, I had to run to my family.” (06, PWUD, Jakarta). Other survival strategies included reducing daily meal consumption or engaging in small business ventures:

Because I was laid off my expenses increased, and my income decreased. It has been dwindling more and more. So yeah, I took an order for mending clothes and then recycling them at the cheapest prices so that I could eat. (01, TGW, Jakarta)

#### Impact on social relationships

All 22 participants reported that adhering to social distancing to avoid contracting COVID-19 coupled with the isolating effects of Indonesia’s public health lock-down negatively affected their social relationships. Social distancing proved highly difficult to observe, especially with family members, friends, or a sexual partner. As one male participant lamented, “The toughest time during this pandemic is the limited time I have to meet with my partner. So, all this time I have just been home alone.” (14, MSM, Jakarta)

Loneliness and isolation from others were frequent consequences of maintaining social distancing. So was the possibility of being resented by close family and friends for refusing to meet in person. Phone calls and online meetings could help in maintaining interpersonal contact, but these were not perceived as emotionally satisfying or socially bonding as an in-person conversation. Yet when participants did manage to get together after an extended hiatus, it was not uncommon to discover that the interpersonal quality of the relationship had deteriorated. They found that fear of contracting COVID-19 and the demands of social distancing could call into question the strength, reciprocities, and/or expectations of the relationship itself:

She was one of my best friends. Before the pandemic, I usually stayed at her house when I had to come home late from work. One day, I asked her to let me sleepover because it was late at night, and I was scared of being mugged on the street. But she refused and said, “I’m afraid of getting COVID.” It’s really sad that she rejected me --- my own best friend! One day, she got COVID, and she asked me to sleepover and said that she missed me so much. I said to myself, “now, when you need me, then you allow me to sleepover.” (03, FSW, Jakarta)

#### Impact on psychosocial and mental health

All 22 participants reported experiencing adverse psychological or mental health symptoms that they attributed to the challenges of life lived amidst a highly infectious pandemic. Psychological response to COVID-19 typically appeared in the form of fear, worry, and/or anxiety over such threats as viral exposure, dwindling personal finances, job insecurity, and uncertainty about prospects for the future.

The physical problem is that I am skinnier, but it’s actually more psychological. It’s harder to sleep worrying about tomorrow, worrying about getting infected with COVID. I feel that I almost have chronic depression. (17, MSM, Jakarta)

Worry about contracting the virus with its social and physical consequences could manifest in such mental and somatic disorders as shortness of breath, headaches, eating disorders, and sleeping problems. A female sex worker from Bali complained, “My headaches have increased during the pandemic.” (15, FSW, Bali). A MSM participant from Bali noted that, “What I experienced the most is insomnia.” (08, MSM, Bali). Meanwhile, interpersonal disagreements and even small frustrations could evoke feelings of anger. One male participant disclosed, “I am now quicker to feel emotional and get angry. Sometimes, merely small things can be so emotional.” (10, MSM, Jakarta). Yet, whom to blame for the situation was unclear as one participant mused:

Activity has decreased, work has decreased, and my income has decreased. Also, I cannot meet relatives in the village. I don’t know who to be angry with. (09, MSM, Bali)

Participants discovered that working from home rather than with others or in the community could prove highly stressful due to government-imposed isolation that was often exacerbated by unreliable communication technology. A community outreach worker described the evolution from initially feeling positive about working from home to the realization that doing so could be very emotionally taxing:

Back in March, we already were working from home. [We thought initially], maybe it would be fun... “OK, we can work from home. We can arrange a work schedule.” But it turns out that after 2 to 3 weeks or a month, it becomes very stressful, really stressful. We haven’t met with our friends; we can’t meet with our family. Everything is coordinated via zoom. With a video call, sometimes there’s a lot of trouble with the signal. (07, MSM, Jakarta).

Adverse health behavior emerged or accelerated during the pandemic in what participants perceived as personal response to COVID-19-related stress and social isolation. Smoking was a common coping mechanism. Other detrimental health behavior included increased food consumption, alcohol binging, and using psychotropic or injection drugs. A chronic drug user explained, “During this pandemic, I messed up… using more amphetamine, marijuana, heroin, anti-depressants.” (09, PWUD, Jakarta). Another participant who regularly engaged in sex work confessed:

With the depression I’ve had [due to COVID], I am aware that I need to drink when I serve my clients. Then I can forget about everything, such as whether we were using a condom or not, because we were drunk. Because of my depression, I need to get drunk and smoke. I am really concerned that I will make a pattern of drinking alcohol or smoking marijuana when I serve clients.” (10, MSM, Jakarta).

#### Impact on HIV Adherence

Participants perceived that, for the most part, they routinely practiced fair to good ARV adherence. In explaining why she rarely missed a dose, a sex worker explained, “I really need the medicine because I depend on my health, so I take my medication.” (12, FSW, Bali). Personal experience with being hospitalized for an HIV complication or losing a close friend or partner to HIV could convince participants of the need to maintain a regular medication schedule. A woman participant recounted, “I often see bad things happening to those who don’t take their ARV (anti-retroviral) medication regularly or who even stop their medication. It happened to someone I knew personally.” (02, TGW, Bali)

Despite best intentions, participants discovered that it could prove exceedingly difficult to maintain consistent adherence during the pandemic. Clinic hours changed for the worse to accommodate reduced staffing. Newly revised pick-up times to refill medication conflicted with many work schedules. One participant complained, “I had to stop taking ARV for three days because there really wasn’t any stock at the clinic.” (01, MSM, Bali)

Participants reported that clinic healthcare workers tried to ameliorate ARV shortages by rationing medication dosages while awaiting the arrival of new supplies or by referring patients to larger hospitals with greater stockpiles. Unfortunately for those who were referred, this solution frequently failed, and they were sent back after only a few weeks. Dispensing expired medication also occurred. A participant related an argument that he had with a service provider over dated medication:

He said it can still be used. But it was clear that the bottle was marked for an expired date on 31st August, while I was given the drugs on 15th August. I told him that the drugs are already expired. The staff is angry at me. They said they only have that stock left. I paid the service fee. I paid for it, yet I was given an expired drug. What if there are negative side effects for patients who take an expired drug? (01, TGW, Jakarta)

The study’s more proactive participants reported finding ways to counter such ARV shortages. Tactics included borrowing the medication from a HIV-positive friend or partner, asking a close associate who worked at a clinic to appropriate the needed ARVs (possibly illegally), rationing unused medication, and going around the health care system. “When I knew there was going to be a shortage of ARVs,” one participant explained, “I immediately bought them online for myself with my own money.” (11, MSM, Bali)

In addition to ARV medication shortages, other impediments to ARV adherence that participants reported included the expense of traveling to a clinic for refills given their reduced incomes, fear of being exposed to COVID-19 during a clinic visit, and medication fatigue.

It’s not that I don’t want to take my ARVs, but I don’t have any. Before the pandemics we already had to pay administration fee and it’s harder now. I need to go to the clinic 3 to 4 times a month, with additional cost for transportation. Therefore, I sometimes decide not to take the medication. I’ll just plan to go to the clinic next month instead. (02, PWUD, Jakarta)

### Social Support during the Pandemic

Clearly coping with the stress and restrictions imposed by the COVID-19 pandemic proved an enormous challenge for PLWH. In the face of high stress and deprivation, it is not unusual for people including our study participants to turn to informal and/or formal sources of support to help meet their emotional, financial, and/or other needs.

#### Informal Sources of Support: Family, Friends, and Key Population Peers

Participants described drawing upon their familial and personal social networks including HIV key population peers for emotional, instrumental, and/or informational support.

*Emotional support* provided by family and close social network relationships consisted of conveying to participants, and sometimes mutually sharing, supportive feelings such as empathy, affect, and reassurance that things will get better. Participants reported feeling less fearful and more hopeful about their lives and future after sharing their problems or worries with someone even if nothing significant changed. One respondent explained, “I tell my friends if there are problems even though sometimes, I don’t find a solution.” (03, FSW, Jakarta)

*Instrumental support* consisted of tangible support such as money, food, material goods, temporary housing, and care when ill. Financial help was the most frequently mentioned form of instrumental support. As one informant confessed, “ I borrow from family sometimes to cover my daily needs. I don’t have any choice.” (06. PWUD, Jakarta). Or as another informant asked rhetorically and then self-answered: “What do you do if you’re in a tight spot? I have to borrow some money from others.” (07, FSW, Bali)

*Informational support* took the form of being given advice or engaging in a mutual sharing of problems and possibly solutions. An informant described the good advice that she received from a peer:

I have a friend in my peer support group who is also from the community who suggested that ‘it’s better for us to sell roasted rice around than do nothing.’ So, I said, “let’s do it! Let’s sell rice around.” Thank God, I was helped and supported by the transgender community. And well, even though I only earn a little, I have had help like that. (01, TGW, Jakarta)

As evidenced by informants’ accounts, family, friends, and members of social networks frequently functioned as crucial sources of informal support, but not always. Participants told of parents or family who lived too far away to be of much help or who had their own daunting challenges to overcome. In some instances, familial relationships were strained and unavailable due to negative judgements about a participant’s behavior such as using illicit drugs, selling sex, or engaging in same sex behavior. As one transgender woman lamented about her family’s rejection of her, “No one cares about me, no one cares.” (01, TGW, Jakarta)

#### Formal Sources of Social Support: Government and Community Organizations

Participants also reported instances of receiving COVID-related formal assistance from the Indonesian government. For example, one participant reported receiving a cash transfer as a “pre-employment allowance” while he searched for work. (Participant 01. MSM, Bali). Local municipalities also provided conditional cash allowances (*bantuan langsung tunai*), food, and other supplies to PLWH who were in dire need. Unfortunately, from recipients’ perspective, the duration of such support could prove far too brief.

I once received rice and a few supplies from a Bekasi City government agency. It was enough for 4–5 days and maybe up to a week. But it was for one time only. When the government gives out these items, it doesn’t think about duration and how long they will last. But I shouldn’t blame the government in the city where I live. I should instead say thank you. (02, PWUD, Jakarta)

NGOs and peer groups organized around advocacy and support for HIV key populations frequently functioned as vital safety nets for their members. Assistance varied but tended to take the form of grocery and food distributions, cash allowances, meals served at food kitchens, and help in applying for public aid. Such formal assistance proved especially valuable to participants who found it difficult-to-nearly impossible on their own to compile the paperwork or documentation needed to establish their eligibility for government support.

### Challenges to Health Service Use

The correlates of the COVID-19 lockdown and mandated social distancing created numerous impediments for participants in accessing not only COVID-19 services but also HIV and other medical care. Financial strain due to diminished income made it especially difficult to meet the medical and transportation costs of accessing medication for HIV. A transgender woman described the difficulties that she faced in accessing the ARV medication that she needed:

One of my barriers to services is the cost constraint. I often borrow from friends for [the necessary clinic] registration fee. I also don’t have the money to go. … That’s why I want money from the government or from PLHIV health services agencies [in the form of] transport compensation or something like that for people like us. There are so many people like me. (06, TGW, Bali)

Similar financial and practical challenges could hinder access and the use of mental health services needed to counter the effects of severe stress, anxiety, sleeplessness, and other debilitating conditions due to COVID-19. While participants who suffered from these disorders expressed the desire to access mental health services, few knew how to do so. One participant explained, “We don’t know about mental health services, so we’re confused.” (10, MSM, Jakarta) “And its expensive,” complained another informant who didn’t know about the availability of community free services. (11, MSM, Bali) In addition, the value of psychological counseling and mental health services were sometimes questioned or not seen as being needed. “In my opinion, it’s not that convincing.” (01, MSM, Bali] “I have my own healing system,” another participant explained. (14, MSM, Jakarta).

Participants were well-aware of the social stigma surrounding HIV and their PLWH status. They feared that contracting COVID-19 would become an additional source of social censure should they acquire it. One participant mused, “The stigma of getting the coronavirus is harsher than getting HIV.” (14, PWID, Bali). Another asked quizzically:

If I died because of COVID, what would happen? …. It’s a sin to die because of COVID and my family would be isolated by the neighborhood. And if I get COVID, I am afraid I will be evicted or something like that. (08, TGW, Jakarta)

In seeking medical and mental health services for COVID, informants feared that they would encounter negative judgements by medical staff because of their HIV status, sexual behavior, drug use, and/or livelihood as a sex worker. Transgender women, for example, uniformly reported great difficulty identifying mental health providers who were sensitive to transgender issues. Engaging in same sex behavior also was seen to potentially evoke provider condemnation. One male participant who has sex with men reported a past instance of negative counseling about his sexuality that continues to hurt him:

When I was in Jakarta, I was exposed for the first time to information about sexuality. So, I tried to see a psychologist for consultation about why I am gay. [I asked] why am I in conflict with myself? Instead of helping me to understand, she judged me instead by saying… “remember that God created men and women. Your nature is as a man, and yes, you must carry out nature. You can’t violate nature like that.” I felt judged and a bit traumatized by that.” (17, MSM, Jakarta)

Yet not all informant encounters with mental health and counseling services turned out bad. A female participant who sold sex for a living reported that, “I feel quite satisfied with the services that I received. The doctor seemed familiar, friendly, and liked to ask and answer questions.” (15, FSW, Bali) Another informant acknowledged that receiving counselling from a professional psychologist helped him to overcome internalized negative judgements about being HIV-positive that drove him to contemplate taking his own life.

It’s fun! I now know my weaknesses and what I should do. I skipped counseling when I found out I had HIV. I would wake up every Saturday morning wanting to kill myself over all kinds of things. I came to know [through counseling] what made me sabotage myself like that. So far, I’m very satisfied. (11, MSM, Bali)

## Discussion

This study is one of the few to examine the impact of the global COVID-19 pandemic on the lived experiences of people living with HIV in Southeast Asia. Its results add to a growing body of findings similarly reported by other COVID-19 studies throughout the globe [3], which are now confirmed as similar for Indonesia. The COVID-19 pandemic undoubtedly has exerted a strong negative impact on the general populace of all countries, but its syndemic effects on people living with HIV adds an additional psychosocial, economic, and clinical burden on the health and well-being of PLWH as an already socially and often financially compromised vulnerable population. The study’s sample of participants drawn from Indonesia’s four HIV key populations represents a broad set of voices recounting a plethora of COVID-19 consequences for this marginalized group.

From a public policy standpoint, the study’s findings point to the many latent consequences of government policies to curb the outbreak of an emerging pandemic through enforced social distancing and stay-at-home orders. Legal bans against traveling freely and policy restrictions against in-person meetings disrupted most regular social activities and employment practices. As reported by study participants, such prohibitions could result in personal stress, anxiety, and symptoms of depression further triggered by loss of connectedness to others and uncertainty as to the future. Coping with these challenges could motivate detrimental health practices such as increased smoking behavior, unhealthy eating habits, and adoption or increase in illicit drug use. Yet when their accounts are viewed overall, participants showed evidence of considerable fortitude and resourcefulness in meeting the many challenges of living within the financial, psychosocial, and physical constraints of a deadly pandemic. Going around the system to get ARVs when unavailable and tapping into informal social support from family, friends, and HIV-positive peers proved essential in assisting them to persevere as did formal support when available through government subsidies and NGO assistance.

Recognizing from the start of the pandemic that a public health lockdown could prove psychosocially devastating to its population, the Indonesian government instituted a free online counseling initiative (SEJIWA) to assist individuals experiencing severe mental health distress. Few participants in this study, however, knew of this resource and none had used it. In addition, the government also instituted a series of cash and food subsidy programs for the poor and recently unemployed. Although it appears from the study’s data that such input could prove a temporary lifeline for PLWH in financial straits, it also tended to be temporary and insufficient in meeting long-term basic subsistence needs. Difficulties in amassing and completing the documentation and paperwork required to successfully apply for government assistance also left some without this formal help. Participants reported that NGOs could prove highly valuable in helping to successfully meet such government requirement for assistance.

Fortunately for the study’s participants, finding the money needed to purchase ARVs was not a problem. The Indonesian government provides free ARV medication for PLWH, and participants reported fair to good adherence in taking it. Yet maintaining consistent adherence could prove highly challenging for Indonesia’s PLWH due to clinics’ limited supplies at the start of the pandemic, medical registration costs that had to be paid to receive free government treatment, and the expenses and added burden of regular clinic visits during the lockdown. Not unlike the experience common with individuals living with HIV prior to COVID-19 [14], medication fatigue also could exert a toll on consistent HIV medication use. Similar challenges to maintaining ARV adherence in Indonesia during the pandemic have been reported in multiple countries including China [15], Mexico [16], Vietnam [1, 17]; and much of Asia [18].

The scientific literature contains numerous recommendations and effective strategies for adoption by countries and health providers to help support ARV adherence during a disease pandemic or period of national crisis. These include instituting access to refills by mail and drive-through refill booths, providing patients with extra medication and extended ARV refills to obviate transportation problems and to tide them over during periods of shortage, heightened coordination between local CDC clinics and hospitals to expand existing refill sites, and mental health online counselling to remove psychological barriers to accessing ARV services[1, 15, 19, 20]

Much of the current body of HIV COVID-19 research reports on the impact of the COVID-19 pandemic during its first year. Since then, other variations of the virus have emerged, public health policies in many countries have shifted, and vaccines and other medications have been developed to prevent or reduce COVID-19-related mortality and morbidity. Additional research is needed to study the impact of the pandemic on PLWH under such evolving circumstances and also studies that investigate and identify factors that predict successful resilience to its effects. Clinical research shows that people who contract COVID-19 are subject to a possible post-infection sequalae of mental and/or physical health disorders. Sometimes referred to as the pandemic’s “long haulers”[21], the long-term effects of post-COVID-19 infection on the health, behavior, and well-being of PLWH beg to be investigated. Participants also spoke of the potentially stigmatizing effects of testing positive for COVID-19 as members of a HIV key population. Both research and effective interventions are needed that mitigate the intersectional stigma for PLWH of having contracted both HIV and COVID-19. Finally, in addition to government subsidies, formal support through NGOs and informal support from family, friends, and peers proved an essential source of help for PLWH during the pandemic. Yet from the paucity of studies reported in the scientific literature, little is known of the impact of providing such support on these key providers.

### Limitations of the study

The results of this research are based on data gained from a convenience sample of 22 HIV-positive participants recruited from 9 HIV NGOs approximately 9 months after the government instituted its lockdown policy. Its findings may not fully generalize to PLWH whom this recruitment method failed to reach or who chose not to participate in the study. Neither do the study’s results necessarily represent what these same participants or other PLWH may have experienced over time as the pandemic unfolded and its effects possibly changed. Also without a comparison sample of people not infected with HIV, it is impossible to know to what extent the study’s findings are unique to the syndemic of living with HIV while confronting the health and psychosocial demands of COVID-19 as an intersecting pandemic. Finally, the analytic categorization of participants that assigned each of them to a particular key population was based on how they self-identified when seeking NGO services. The study’s initial data analysis sought to compare similarities and differences in COVID-19 experience between the study’s four key population groups. While potentially informative, such analyses proved impossible due to overlapping behavior. Engaging in illicit drug use and commercial sex was not uncommon among members of all four populations, and same sex behavior was not solely confined to men who self-identified as MSM.

## Conclusions

In May 2023, the World Health Organization announced that COVID-19 no longer constitutes a public health emergency of international concern although remaining a global health threat [22]. As with numerous other countries, the pandemic in Indonesia appears to be substantially waning as newly developed vaccines against infection exert their effects[6] Nonetheless, mutations of dangerous new variations of COVID-19 remain a constant threat. Also, over the last 50 years, the world increasingly has seen the emergence and reemergence of viral and bacterial pandemics that pose serious mortality and morbidity threats [23]. In addition to HIV, these include ZIKA, SARS (Severe Acute Respiratory Syndrome), MERS (Middle East Respiratory Syndrome), Ebola, and Disease X (a yet unknown future pathogen). Although seemingly effective epidemiologically, the results of this study point to the latent consequences of government attempts to curb the spread of a pandemic through public health lockdowns and enforced policies of physical separation between citizens. The participants’ experiences are vivid reminders of the importance of ensuring that public safety nets for PLWH are available if needed that supplement and assist more informal sources of instrumental and psychosocial support provided by family, friends, and others in the community.

## Figures and Tables

**Table 1:** Demographic characteristics of participants by key population (N = 22)

Variable		MSM (n = 9)	FSW (n = 5)	TGW (n = 4)	PWUD (n = 4)	Total
		n (%)	n (%)	n (%)	n (%)	n (%)
Age in years	18–28	1 (4.5)		1 (4.5)		2 (9.1)
	29–39	6 (27.3)	4 (18.2)	3 (13.6)	4 (18.2)	17 (77.3)
	> 40	2 (9.1)	1 (4.5)			3 (13.6)
Gender	Male	9 (40.9)			4 (18.2)	13 (59.1)
	Female		5 (22.7)			9 (40.9)
	Transgender woman			4 (18.2)		
Educational Background	Elementary School	1 (4.5)		1 (4.5)		2 (9.1)
	Junior High School		2 (9.1)	2 (9.1)		4 (18.2)
	Senior High School	4 (18.2)	3 (13.6)	1 (4.5)	4 (18.2)	12 (52.5)
	Undergraduate	4 (18.2)				4 (18.2)
Occupation	NGOs related to HIV	4 (18.2)	1 (4.5)	1 (4.5)	1 (4.5)	7 (31.8)
	Private sector	3 (13.6)			1 (4.5)	4 (18.2)
	Self-employed	1 (4.5)	4 (18.2)	2 (9.1)		7 (31.8)
	Unemployed	1 (4.5)		1 (4.5)	2 (9.1)	4 (18.2)
Residency	Jakarta (and suburban area)	4 (18.2)	2 (9.1)	2 (9.1)	4 (18.2)	12 (52.5)
	Bali	5 (22.7)	3 (13.6)	2 (9.1)		10 (45.5)

MSM = Men Sex with Men; FSW = Female Sex Worker; TGW = Transgender Woman; PWUD = People Who Use Illicit Drugs.
